# IGNITE Status Epilepticus Survey: A Nationwide Interrogation about the Current Management of Status Epilepticus in Germany

**DOI:** 10.3390/jcm11051171

**Published:** 2022-02-22

**Authors:** Christina M. Kowoll, Matthias Klein, Farid Salih, Gereon R. Fink, Henning R. Stetefeld, Oezguer A. Onur, Michael P. Malter, on behalf of the IGNITE Group

**Affiliations:** 1 Department of Neurology, Faculty of Medicine, University Hospital Cologne, University of Cologne, Kerpener Str. 62, 50937 Cologne, Germany; G.R.Fink@fz-juelich.de (G.R.F.); henning.stetefeld@uk-koeln.de (H.R.S.); oezguer.onur@uk-koeln.de (O.A.O.); Michael.malter@uk-koeln.de (M.P.M.); 2 Department of Neurology, Ludwig-Maximilians University Munich, Marchioninistr.15, 81377 Munich, Germany; matthias.klein@med.uni-muenchen.de; 3 Charité—Universitätsmedizin Berlin, Campus Virchow-Klinikum, Klinik für Neurologie, Augustenburgerplatz 1, 13353 Berlin, Germany; farid.salih@charite.de; 4 Cognitive Neuroscience, Research Center Jülich, Institute of Neuroscience and Medicine (INM-3), Leo-Brandt-Str., 52428 Jülich, Germany

**Keywords:** status epilepticus, survey, continuous EEG (cEEG), anticonvulsants, neurological/neurosurgical intensive care unit NICU, neurocritical care

## Abstract

We aimed to evaluate the current management of status epilepticus (SE) in intensive care units (ICUs) in Germany, depending on the different hospital levels of care and the ICU specialty. We performed a nationwide web-based anonymized survey, including all German ICUs registered with the German Society for Neurointensive and Emergency Care (Deutsche Gesellschaft für Neurointensiv- und Notfallmedizin; DGNI). The response rate was 83/232 (36%). Continuous EEG monitoring (cEEG) was available in 86% of ICUs. Regular written cEEG reports were obtained in only 50%. Drug management was homogeneous with a general consensus regarding substance order: benzodiazepines—anticonvulsants—sedatives. Thereunder first choice substances were lorazepam (90%), levetiracetam (91%), and propofol (73%). Data suggest that network structures for super-refractory SE are not permeable, as 75% did not transfer SE patients. Our survey provides “real world data” concerning the current management of SE in Germany. Uniform standards in the implementation of cEEG could help further improve the overall quality. Initial therapy management is standardized. For super-refractory SE, a concentration of highly specialized centers establishing network structures analogous to neurovascular diseases seems desirable to apply rescue therapies with low evidence carefully, ideally collecting data on this rare condition in registries and clinical trials.

## 1. Introduction

Although status epilepticus (SE) represents a life-threatening manifestation of seizures with high mortality and morbidity [1], there are still several unsolved issues concerning its management. For Germany, it is unclear to what extent the existing guidelines, published by the German Neurological Society (Deutsche Gesellschaft für Neurologie, DGN [2]), are followed in everyday clinical practice, especially because not all critically ill patients with SE are treated on intensive care units (ICUs) with neurologists as the patient-managing specialty.

Although continuous EEG recordings (cEEG) in ICUs have become widely available for therapy monitoring in North America recently [3,4], their current use in German ICUs remains to be assessed with to date only one publication from Germany touching the topic of cEEG in neuro-intensive care management [5].

Accordingly, this study aimed to evaluate the current ICU management of SE in Germany concerning the preferences for monitoring and treatment, depending on the different hospital levels of care and patient-managing specialty.

## 2. Materials and Methods

We performed a nationwide web-based survey. The study was outlined and discussed during the regular meetings of IGNITE (Initiative for German NeuroIntensive Trial Engagement), a subdivision of the German Society for Neurointensive and Emergency Care (Deutsche Gesellschaft für Neurointensiv- und Notfallmedizin, DGNI), which represents a free alliance of German neurologists and neurosurgeons on neurointensive care units to conduct multicenter clinical trials in neurocritical care.

Questionnaire: The survey was built and analyzed using SurveyMonkey (surveymonkey.de), a web-based survey platform. The questionnaire consisted of 49 questions with a processing time of about 15 min for each respondent covering the following categories: size, location, and care level of the participating center, characteristics of the ICU, EEG recording, therapy standards, ranking of drugs at the different stages of SE, application of non-pharmacological therapies for refractory cases, therapy goals and network structures between hospitals. The complete questionnaire is available as a Supplementary File. Respondents remained anonymous; no patient data were requested.

Participants: Addressees were physicians in hospitals specialized in neurointensive care management recruited from the website of the DGNI. We searched the corresponding clinic homepages to identify the attending physician responsible for the ICU to contact him/her directly. A link was sent to the attending physician or—if not identifiable—to the head of the department. If necessary, reminders were sent after 4 and 12 weeks. In the case of several departments treating SE patients within one hospital, e.g., in hospitals with both a neurological and neurosurgical ICU, every department was asked to answer separately. Therefore, we refer to the term “department” instead of “hospital” in the following. Financial compensation was not provided. Responses were received between November 2019 and March 2020.

Subgroup analysis: To investigate management differences according to the departmental level and the specialty of the ICU, we compared university departments (UDs) with all other departments (Non-UDs) and ICUs under neurological or neurosurgical responsibility (NICU) to ICUs under the responsibility of other specialists (Non-NICU).

Data Analysis: Statistical analyses were performed using SPSS 26.0 for Windows (IBM, Armonk, NY, USA). As normal distribution was not applicable for any metric variables, differences were tested using the nonparametric Mann-Whitney U test (“#”, UD vs. Non-UD, NICUs vs. Non-NICUs). Differences in nominal and ordinal variables were tested by the Chi-square test (“†”) or Fisher’s exact test (“‡”, if less than five items). For more than 2 × 2 fields, the asymptotic significance was determined, and the single comparisons were analyzed by paired difference test via Z tests and the Bonferroni correction (“+”). All tests were performed two-tailed. *p*-values < 0.05 were estimated to be significant. Within the text and figures/tables, *p*-values are accompanied by the symbols in parenthesis mentioned above to indicate the applied statistical method to calculate them.

## 3. Results

### 3.1. Participants and Number of Cases

We identified and contacted 232 departments, and 83 departments participated (36%). The number of respondents is given in presenting the individual results if not all respondents answered the respective question. Of the participating 83 departments, 36 (43%) were located at UDs and 47 at Non-UDs (57%). When dividing participating departments into groups concerning the specialty of the ICU in terms of department association, 38 were NICUs (46%), and 45 were Non-NICUs (54%).

UDs treated significantly more neurological patients per month than Non-UDs (”>30 neurological patients“: UD 20/36, 56% vs. Non-UD 12/47, 26%; *p* = 0.005 (+). Remarkably, the estimated number of SE patients, status types, and treatment responsiveness did not differ among levels of care or ICU specialty. Further details on the characteristics of the participating departments are given in Table 1.

### 3.2. EEG Diagnostics

**Figure 1:**
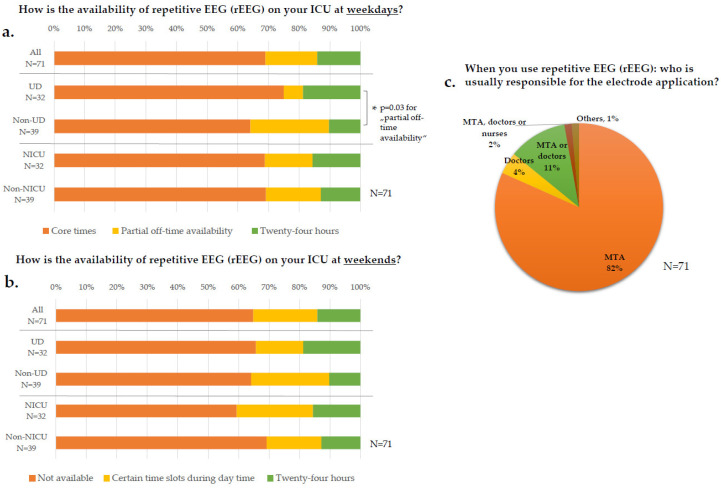
**EEG diagnostics.** (**a**,**b**): Participants had to choose one out of three possible answers marked by different colors; upper bars: all participants answering (“all“); 2nd and 3rd bar: answers of “all“ subdivided according to the level of care, 4th and 5th bar: according to ICU specialty. (**c**) Multiple answers were possible; there were no significant differences regarding department level of care or ICU specialty; MTA= medical technical assistant. Data are displayed as percentage of survey participants; N number of respondents; * = significant; UD = university department; Non-UD = Non-university departments; NICU = neurological/neurosurgical ICU; Non-NICU = non-neurological/neurosurgical ICU; ICU: intensive care unit; *p*-value: was determined by paired difference test via Z tests and the Bonferroni correction.

On weekdays, the availability of repetitive EEG (rEEG) recordings was mainly limited to core working hours in 49/71 ICUs (69%) (Figure 1a). On weekends, however, rEEG was not available in most ICUs (46/71, 65%) (Figure 1b), without any difference depending on the department level of care or specialty of the ICU.

**Figure 2:**
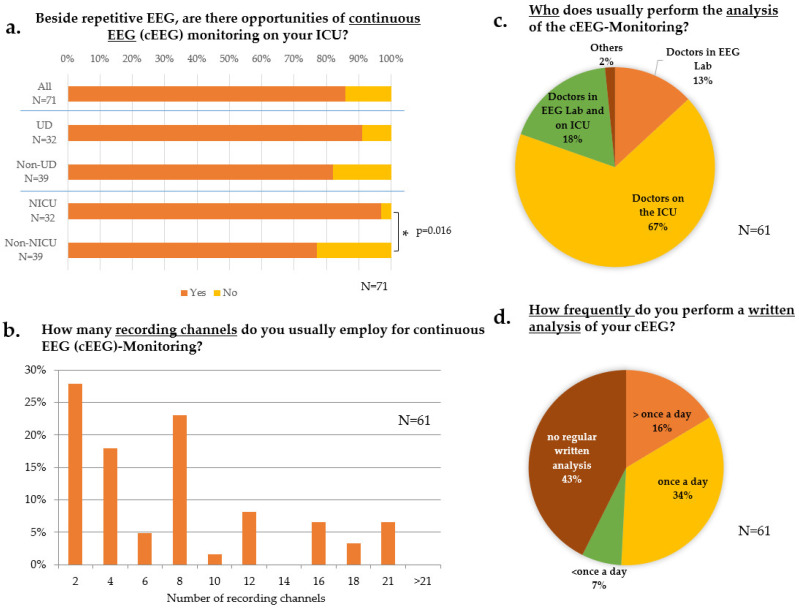
Continuous EEG monitoring (cEEG). (**a**) Upper bar: all participants answering the question (“all“); 2nd and 3rd bar: “all“ subdivided according to the level of care; 4th and 5th bar subdivided according to specialties; * = significant. (**b**) Number of recording channels for continuous EEG (cEEG). (**c**) Personnel performing the analysis of cEEG; participants had to choose one out of four answers. (**d**) Written analysis of cEEG; participants had to choose one out of four answers. Data are displayed as percentage of participants answering this question. N number of respondents, UD = university department; Non-UD = Non-university departments; NICU = neurological/neurosurgical ICU; Non-NICU = non-neurological/neurosurgical ICU; ICU = intensive care unit; EEG = electroencephalography; *p*-value was determined by the Chi-square test.

CEEG was available in most responding departments (61/71, 86%), equally distributed across UDs and Non-UDs. Concerning the ICU’s specialty, cEEG was implemented more frequently on NICUs than on Non-NICUs (31/32, 97% vs. 30/39, 77%; *p* = 0.016‡, Figure 2a). The cEEG implementation was heterogeneous across the ICUs concerning the number of utilized recording channels (Figure 2b) without significant differences regarding department level of care or ICU specialty.

CEEG analysis was commonly performed visually (53/61, 87%). Besides, only 8/61 (13%) ICUs used quantitative analysis tools additionally. None of the respondents relied on automated analyses alone. No differences regarding department level of care or ICU specialty were noted.

The interpretation of cEEG was exclusively conducted by physicians of the ICU team in 41/61 (67%) of ICUs (Figure 2c). At Non-UDs, a higher proportion of the cEEGs was interpreted by physicians of the EEG laboratory than at UDs (UDs 0/29, 0% vs. Non-UDs 8/32, 25%, *p* = 0.004+). No significant difference was noted among specialties. However, in only 31/61 ICUs (51%), a written cEEG report was obtained at least once a day. In 26/61 ICUs (43%), no written documentation of cEEG analysis was performed regularly (Figure 2d). The latter result was independent of the departmental level of care or specialty of the ICU. Technical requirements for simultaneous video-EEG-monitoring were disposable in 14/61 ICUs (23%), with no difference among department level of care or specialty of the ICU. Tools for external EEG analysis for the supervising physician existed in only 9/71 departments (13%). There was no difference among department level of care or specialty of the ICU.

### 3.3. Therapy

#### 3.3.1. Standard Operating Procedures (SOP)

While 31/69 (45%) of the respondents stated to adhere to an obligatory internal SOP for the management of SE, 12/69 (17%) had an SOP with only partial adherence. Of the ICUs, 26/69 (38%) did not have an SOP, but 23/69 (33%) of them stated following similar approaches, and in only 3/69 (4%) management decisions were made individually. No differences regarding department level of care and specialty of ICU were found on this topic.

#### 3.3.2. Substance Preferences and Sequences

**Figure 3:**
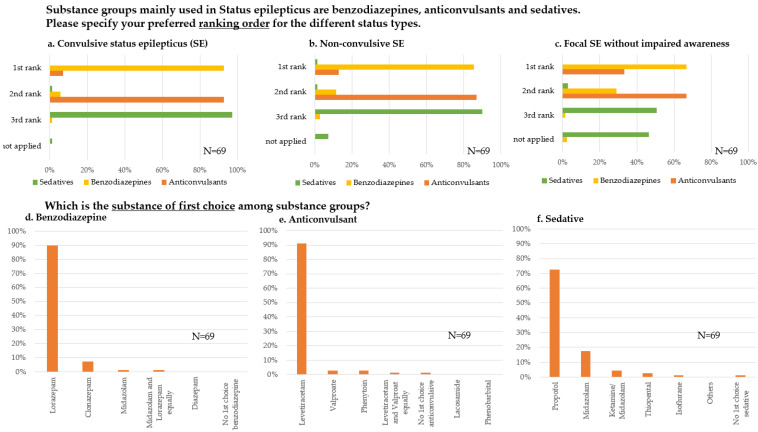
Pharmacotherapy in status epilepticus (SE). (**a**–**c**) Ranking order of substance groups in convulsive SE (**a**), non-convulsive SE (**b**) and focal SE (**c**). (**d**–**f**) Substance of first choice among benzodiazepines, anticonvulsants and sedatives. Data are displayed as percentage of survey participants answering this question. N = number of respondents; SE = status epilepticus.

We noted a general agreement regarding the ranking order of substance groups from the initial to the refractory phase of generalized convulsive SE: benzodiazepines on rank 1 (64/69, 93%), anticonvulsants on rank 2 (64/69, 93%), and sedatives on rank 3 (67/69, 97%) (Figure 3a). A similar distribution was found for the treatment of non-convulsive SE (Figure 3b) with the difference that Non-UDs and Non-NICUs stated not to use sedatives (“not applied”: UD: 0/32 (0%) vs. Non-UD: 5/37 (14%), *p* = 0.03+; NICU: 0/31 (0%) vs. Non-NICU: 5/38 (13%), *p* = 0.04+). In the case of focal SE without impaired awareness, even more respondents stated to use anticonvulsants in the first place (23/69, 33%, Figure 3c). No differences regarding department level of care and specialty of ICU were observed.

When asked about the particular substance preferences, the first choice for benzodiazepines was mostly lorazepam (62/69, 90%, Figure 3d), for anticonvulsants levetiracetam (63/69, 91%, Figure 3e), and for sedatives propofol (50/69, 73%, Figure 3f), equally answered among department level of care or specialty of the ICU.

**Figure 4:**
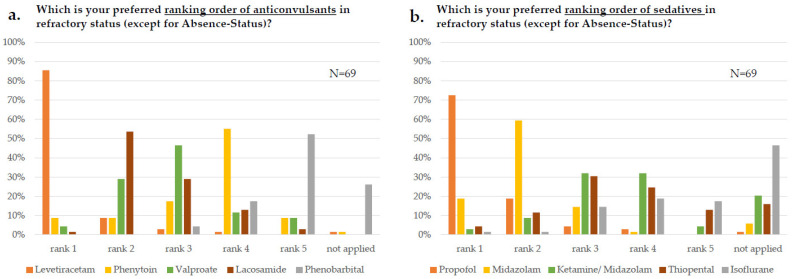
Anticonvulsants and sedatives in status epilepticus (SE). (**a**) Ranking order of anticonvulsants (**b**) Ranking order of sedatives. Participants were asked to put the five substances marked by different colors into a ranking order. Multiple answers were not allowed. Data are displayed as percentage of survey participants answering this question. N = number of respondents.

In the case of sequential use of anticonvulsants in refractory SE, accordingly, there was a clear preference for levetiracetam at the first rank (59/69, 86%, Figure 4a). Lacosamide was chosen as the most frequent second rank anticonvulsant (37/69, 54%); at third rank, valproate was preferred (32/69, 46%), whereas phenytoin (38/69, 55%) and phenobarbital (36/69, 52%) were preferred at rank 4 and 5, respectively. When asking for a ranking order for sedatives, there was an overall agreement for propofol as the first rank drug (50/69, 73%), followed by midazolam on rank 2 (41/69, 59%). After that, no clear preferences for any substance were found (Figure 4b). Isoflurane was more frequently used at UDs compared to Non-UDs (“not applied”: UD: 10/32 (31%) vs. Non-UD: 22/37 (60%), *p* = 0.019+) and even more frequently on NICUs than Non-NICUs (“not applied”: NICUs 9/31, 29% vs. Non-NICUs 23/38, 61%; *p* = 0.009+). Among ICU specialties, ketamine/midazolam was more frequent in NICUs than Non-NICUs (30/31, 97% vs. 25/38, 66%, *p* = 0.002+).

**Figure 5:**
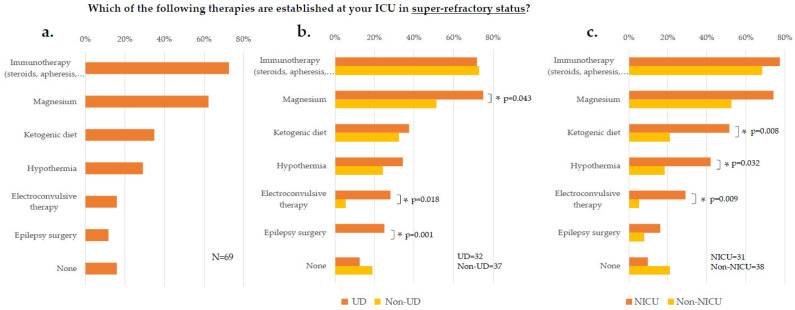
Further therapies in super-refractory status. Answered by all participants (**a**), among levels of care (**b**), and among specialties (**c**). Data are displayed as percentage of survey participants answering this question. N number of respondents, UD = university department; NICU = neurological/ neurosurgical ICU; ICU = intensive care unit; * = significant; *p*-values were determined by Chi-square test or Fisher’s exact test.

Beyond sedatives and anticonvulsants, 58/69 (84%) of participants applied further therapies in refractory and super-refractory stages of SE, thereunder most frequently immunotherapy (50/69, 73%) and magnesium (43/69, 62%, Figure 5a). The following strategies were more often used at UD than Non-UDs: magnesium (24/32, 75% vs. 19/37, 51%; *p* = 0.043†), electroconvulsive therapy (9/32, 28% vs. 2/37, 5%; *p* = 0.018‡), and epilepsy surgery (8/32, 25% vs. 0/37, 0%, *p* = 0.001‡; Figure 5b). Differences between NICUs and Non-NICUs were noted regarding ketogenic diet (16/31, 52% vs. 8/38, 21%; *p* = 0.008†), hypothermia (13/31, 42% vs. 7/38, 18%; *p* = 0.032†), and electroconvulsive therapy (9/31, 29% vs. 2/38, 5%; *p* = 0.009‡; Figure 5c).

**Figure 6:**
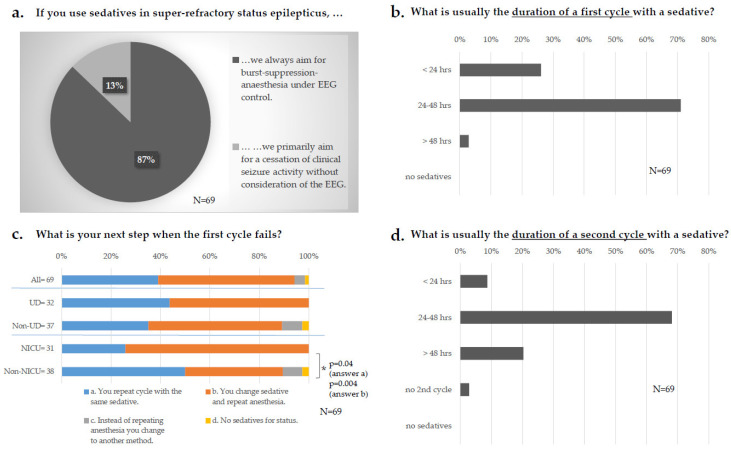
Super–refractory status. (**a**) Therapy goal under sedatives. (**b**) Duration of the first cycle with a sedative. (**c**) Next step after failure of the first cycle: upper bar: all participants answering the question (“all“); 2nd and 3rd bar: “all“ subdivided according to the level of care; 4th and 5th bar subdivided according to specialties. (**d**) Duration of the second cycle with a sedative. Data are displayed as percentage of survey participants answering this question. N = number of respondents, UD = university department; NICU = neurological/ neurosurgical ICU; hrs = hours; * = significant; p-values were determined by paired difference test via Z tests and the Bonferroni correction; EEG = electroencephalography.

When asked about therapy goals under sedatives, 61/69 (87%) of the respondents stated that they primarily aimed for burst-suppression-anesthesia (Figure 6a). The first cycle with a sedative was set mainly between 24 and 48 h by 49/69 (71%) respondents (Figure 6b) without any differences in the level of care or specialties. If the first cycle of sedatives failed to terminate the SE, 38/69 (55%) stated changing the sedative and repeating anesthesia (Figure 6c). This approach was more common on NICUs than on Non-NICUs (23/31, 74% vs. 15/38, 40%; *p* = 0.004+), whereas Non-NICUs preferred to repeat the cycle with the same sedative (19/38, 50% vs. 8/31; 26%; *p* = 0.04+). When respondents decided for a second cycle with a sedative, the stated duration was between 24 and 48 h in 47/69 (68%) cases and in 14/69 (20%) longer than 48 h (Figure 6d), not differing among levels of care or specialties.

### 3.4. Network Structures and Established Follow-Up Evaluation

52/69 (75%) of the participants stated not to transfer patients with refractory or super-refractory SE to other departments. In only one ICU (1.4%), standardized transfer structures existed analogous to neurovascular emergencies. Non-UDs transferred more often compared to UDs (Non-UD 14/37 (38%) vs. UD 2/32 (9%), *p* = 0.002+), the same was true for Non-NICUs compared to NICUs (Non-NICU 13/38 (34%) vs. NICU 4/31 (13%), *p* = 0.016+).

After discharge, follow-up interviews were performed in 39/69 (57%) of participating ICUs, thereunder only at 12/69 (17%) regularly and at 27/69 (39%) on an irregular basis without differences regarding departmental level of care and specialty of ICU.

## 4. Discussion

This study is the first national survey that provides “real world data“ concerning the current management of SE at different levels of care in Germany. The main findings were that: (i) In almost 50% of responding departments, patients are treated at an ICU specialized in neurology or neurosurgery; (ii) Most ICUs who took part in our survey can use continuous EEGs; and (iii) High agreement among respondents was found about the medical treatment of SE patients, with management differences in the level of care and specialty of ICU being low.

### 4.1. Participating Departments

In Germany, in the past, investigator-initiated clinical studies were mostly performed at university sites, neglecting many non-university departments where patients are cared for in everyday practice. To avoid this selection bias, we invited clinicians from departments of all levels of care to participate in our survey. Consequently, 57% of the participating departments were not located at university hospitals. Furthermore, in Germany, specialized ICUs attached to neurological departments with neurologists in the lead are not widely established, i.e., many neurological emergencies are managed at non-NICUs. This aspect of general health service is reflected by more than half of the participating ICUs (54%) treating SE patients that are not attached to neurological/ neurosurgical departments. Thus, the management of SE is an interdisciplinary issue in Germany. Given these circumstances, an overall response rate of 36% of the initially addressed departments seems satisfactory.

### 4.2. EEG Diagnostics

Whereas cEEG in ICUs has become a widely established tool for SE therapy monitoring in the US [3,4], a recently published nationwide survey from the Netherlands, in which 78% of all hospitals with a neurological department and ICU participated, reported a cEEG in 36% [6]. Among specialties, patients at NICUs underwent cEEG more frequently. In a recent study from Switzerland, cEEG led to increased seizure detection and treatment modification but was not related to improved outcome compared with rEEG [7]. This suggests that cEEG can be replaced by rEEG if the latter is performed frequently and with appropriate expertise.

Despite its widespread availability, the implementation of cEEG was highly heterogeneous among the respondents. Although at least 16 channels according to the 10–20-system are recommended in the current guidelines of the American Clinical Neurophysiology Society [8,9], this was followed only in 10/61 (16%) participating departments. In Germany, explicit and mandatory recommendations for using cEEG in critically ill patients are currently not provided by the German Society for Clinical Neurophysiology and Functional Imaging (Deutsche Gesellschaft für Klinische Neurophysiologie und Funktionelle Bildgebung, DGKN, www.dgkn.de, last accessed on 29 January 2022). Only 23% of responding departments stated to have simultaneous video EEG recording facilities, much less than reported from the US, where it was available in 86% of institutions with cEEG [4]. This discrepancy likely reflects different organizational structures. The job description of a neurophysiologist, exclusively responsible for neurophysiological diagnostics, does not exist in the same way in Germany as in the US. Rather, the head of an EEG laboratory is an experienced clinician.

A critical disadvantage of cEEG is its high demand for technical and human resources. This drawback is reflected in our survey because written cEEG analyses were only established in 50% of the responding ICUs, whereas in 43% of the ICUs, no written report was available regularly. These findings are consistent with a Dutch survey [6], whereas a higher rate of written reports was reported from the US, possibly due to the above-mentioned different organizational standards [3]. Further, in our study, 67% of analyses were only performed by the ICU physician team and not by physicians from the EEG laboratory. EEG analysis mainly performed by the ICU physicians raises concerns about report quality, especially as the diagnosis of non-convulsive SE is challenging [10], and the misinterpretation of EEG by less experienced readers is a well-known pitfall causing potentially harmful over-therapy [11,12].

Only 13% of respondents stated to use quantitative analysis systems, whereas they were used by one-third in the two US surveys [3,4], indicating a more conservative approach in Germany. Implementing quantitative analysis tools might help reduce the high human resources demand of cEEG analysis, e.g., empowering nurse staff to participate in seizure detection [13].

Only 16% of the responding ICUs with cEEG had telemonitoring facilities implemented. This finding is at odds with telemonitoring facilities in 57% of Dutch hospitals [6] and 95% of the US hospitals [4]. Several issues may underlie this discrepancy. Although it is common to store the patient’s records and EEG recordings electronically, remote access to the clinical recording system is usually not provided, often for data security reasons. Furthermore, ICU physicians at night are often interns rather than experienced or certified EEG readers. Future studies are warranted to investigate this critical issue further.

### 4.3. Therapy

Of note, this survey was conducted before the new German SE guideline was published in 2020 [2]. Thus, formally the guideline from 2012 was valid [14]. There was an overall agreement with the former (and current) guideline recommendations concerning the sequential use of benzodiazepines—anticonvulsants—anesthetics in SE [2] among responding departments. No differences between departmental levels of care or ICU specialty for the initial, established, and refractory stages of SE were detected.

Lorazepam was the benzodiazepine of the first choice, consistent with published evidence at the initial stage of SE [15,16]. In a recent study from SENSE, a multicenter registry recruiting SE patients from eight large hospitals in three German-speaking countries, the proportion of clonazepam was higher than in our survey [17]. This difference may reflect prehospital treatment included in the SENSE study since lorazepam is often unavailable on ambulance service due to a lack of cooling facilities.

Interestingly, the far most preferred first choice anticonvulsant by the responding departments was levetiracetam, despite being only listed as “alternative” anticonvulsant in the National Guideline from 2012. Therefore, the respondents already anticipated the current guideline recommendations [2]. This reflects the growing evidence on levetiracetam’s efficacy while ignoring the still lacking legal approval for its use in SE.

Our study confirms and extends the results of two recent European studies [18,19] on SE treatment. In a monocentric study from Switzerland, levetiracetam was administered in 50% of SE episodes and lacosamide in 40% of SE episodes. However, the sequence preferences remained unclear [18]. Our study could specify that lacosamide was not deemed the first choice anticonvulsant (1%) but was the most frequent second choice (54%) after the failure of levetiracetam (1st rank in 86% of cases). The “classical” drugs phenytoin, phenobarbital, and valproate were less favored by the respondents although still represented in current national guidelines [2]. Further, recent prospective studies have proven comparable efficacy between “classical” and newer drugs [20].

The lesser application of phenobarbital, phenytoin, and valproate likely reflects their metabolic interactions, adverse effects, and safety issues. Of note, a monocentric registry study suggested higher degrees of refractory SE following the use of levetiracetam and lacosamide, probably caused by lower efficacy of the newer drugs [18]. A possible explanation could be habituation to levetiracetam neuromodulatory effects [21]. Further investigations needed on this issue are warranted.

In refractory stages, propofol was the anesthetic of the first choice in the responding departments (71%), midazolam was chosen second after failure. Generally, the level of evidence for the treatment of refractory SE with anesthetics is low. The current National guidelines equally recommend propofol and midazolame [2]. The preference for propofol observed in our study may reflect its favorable pharmacokinetics as coma duration per se is associated with additional complications [22,23]. Immunotherapy was used as the most frequent supportive therapy, reflecting the growing awareness about autoimmune encephalitides in the ICU management [24]. Nevertheless, specific management recommendations regarding suspected autoimmune-mediated SE are lacking.

As super-refractory SE carries a high mortality rate of approx. 35% [25], it seems reasonable to apply rescue therapies even at low evidence. However, these therapies should be evaluated in specialized centers taking part in data surveillance, enabling advances in this field.

### 4.4. Organizational Structures

Regarding organizational/network structures, 75% of participants stated not to transfer SE-patients to another hospital with a significant difference between UDs and non-UDs and between NICUs and non-NICUs. These findings were expected as UDs treated significantly more neurological patients and involved more specialists needed for specific treatments in super-refractory SE, e.g., epileptic surgery or electroconvulsive therapy. Additionally, network structures comparable to stroke care with predefined transfer structures to specialized stroke units do not exist.

### 4.5. Limitations

Our study has several limitations. First, we have to assume a reporting bias. To achieve the highest possible return, we focused on departments listed on the homepage of the DGNI as we assumed that the responsible physicians might be interested in moving the subject forward and would therefore participate. Although we consider a 36% response rate satisfactory in this setting, the participants may not represent the complete spectrum of ICUs treating SE patients. Second, we cannot exclude “desirable” answering and could not verify the response accuracy. Third, not all questionnaires were answered thoroughly. Missing data are likely to have affected the statistical validity of the data.

## 5. Conclusions

SE management in Germany is an interdisciplinary issue throughout different levels of care and with different specialties involved. Therapy monitoring with cEEG seems to be increasingly established, but implementation standards remain heterogeneous. In contrast, the therapy management reflects a homogeneous therapeutic approach from the initial to the refractory phase of SE. For super-refractory SE and rescue-therapies with insufficient evidence, a referral of the patients to highly specialized centers seems desirable. Overall, our results provide evidence that there is room for improvement in the management of SE in Germany.

## Figures and Tables

**Table 1:** Characteristics of the participating departments and ICUs.

	All (*N* = 83)	Uni-Department (UD, *N* = 36)	Non-Uni-Department (Non-UD, *N* = 47)	Sig	NICU (*N* = 38)	Non-NICU (*N* = 45)	Sig
**ICU patient population (*N*, %)**							
Only neurological	29 (35%)	17 (47%) *	12 (26%) *	*p* = 0.040 +	28/38 (74%)	1/45 (2%) *	*p* < 0.001 +
Only neurosurgical	2 (2%)	2 (6%)	0 (0%)	n.s.	2/38 (5%)	0/45 (0%)	n.s.
Interdisciplinary	52 (63%)	17 (47%) *	35 (75%) *	*p* = 0.011 +	8/38 (21%)	44/45 (98%) *	*p* < 0.001 +
	*N* = 83	*N* = 36	*N* = 47		*N* = 38	*N* = 45	
**Specialty of ICU (department association, *N*, %)**							
Neurology	34 (41%)	20 (56%) *	14 (30%) *	*p* = 0.018 +	34/38 (90%) *	0/45 (0%) *	*p* < 0.001 +
Neurosurgery	4 (5%)	4 (11%) *	0 (0) *	*p* = 0.019 +	4/38 (11%) *	0/45 (0%) *	*p* = 0.026 +
Anesthesiology	15 (18%)	1 (3%) *	14 (30%) *	*p* = 0.002 +	0/38 (0%) *	15/45 (33%) *	*p* < 0.001 +
Internal Medicine	8 (10%)	2 (6%)	6 (13%)	n.s.	0/38 (0%) *	8/45 (18%) *	*p* = 0.006 +
Interdisciplinary	22 (27%)	9 (25%)	13 (28%)	n.s.	0/38 (0%) *	22/45 (49%) *	*p* < 0.001 +
	*N* = 83	*N* = 36	*N* = 47		*N* = 38	*N* = 45	
**Median ICU bed size (range)**	12 (5–40)	12 (6–35)	12 (5–40)	n.s.	12 (5–28) *	16 (6–40) *	*p* = 0.002 #
	*N* = 83	*N* = 36	*N* = 47		*N* = 38	*N* = 45	
**Number of neurological patients per month (*N*, %)**							
**<1–10**	22 (27%)	3 (8%) *	19 (40%) *	*p* = 0.001 +	0 (0%) *	22 (49%) *	*p* < 0.001 +
**11–30**	29 (35%)	13 (36%)	16 (34%)	n.s.	13 (36%)	16 (34%)	n.s.
**>30**	32 (39%)	20 (56%) *	12 (26%) *	*p* = 0.005 +	25 (66%) *	7 (16%) *	*p* < 0.001 +
	*N* = 83	*N* = 36	*N* = 47		*N* = 38	*N* = 45	
**Estimated number of SE in 2018 per department (median *N*, range)**	35 (3–250)	45 (3–250)	30 (4–150)	n.s.	50 (3–200)	30 (4–250)	n.s.
	*N* = 71	*N* = 32	*N* = 39		*N* = 32	*N* = 39	
Estimated percentage of SE types in 2018 (median *N*, range)							
Generalized	30 (1–85)	30 (5–80)	25 (1–85)	n.s.	30 (5–80)	30 (1–85)	n.s.
Non-convulsive	40 (5–90)	40 (20–85)	40 (5–90)	n.s.	42.5 (20–90)	40 (5–85)	n.s.
Focal	20 (0–50)	17.5 (0–50)	20 (0–50)	n.s.	19.5 (0–40)	20 (0–50)	n.s.
Absence	1 (0–20)	0.5 (0–20)	1 (0–20)	n.s.	2 (0–20)	0 (0–20)	n.s.
	*N* = 71	*N* = 32	*N* = 39		*N* = 32	*N* = 39	
**Estimated frequency of SE stages in percent in 2018 (median *N*, range)**							
Responsive	40 (10–80)	40 (10–70)	40 (0–80)	n.s.	40 (0–80)	40 (8–80)	n.s.
Established	30 (5–62)	30 (10–60)	25 (5–62)	n.s.	30 (5–60)	30 (10–62)	n.s.
Refractory	15 (4–60)	15 (5–60)	20 (4–60)	n.s.	15 (5–55)	20 (4–60)	n.s.
Super-refractory	10 (0–50)	5 (0–50)	10 (0–50)	n.s.	10 (0–50)	10 (0–30)	n.s.
	*N* = 71	*N* = 32	*N* = 39		*N* = 32	*N* = 39	

3rd and 4th column: descriptive differences between University departments (Uni-department, UD) and Non-university departments (non-Uni-department, Non-UD), significance values in column 5; 6th and 7th column: descriptive differences between neurological/neurosurgical ICU (NICU) and non- neurological/neurosurgical ICU (Non-NICU), significance of differences in column 8; N number of respondents, n.s. non significant, Sig Significance, ICU intensive care unit, SE status epilepticus; * = significant, *p* = *p*-value; #: Mann-Whitney U test; +: Z test and Bonferroni correction.

## Data Availability

The data presented in this study are available on request from the corresponding author.

## Supplementary Materials

The following are available online at https://www.mdpi.com/article/10.3390/jcm11051171/s1, Supplemental File S1: Survey Status epilepticus Germany-Questionnare.
