# Supplementary material for: IGNITE Status Epilepticus Survey: A Nationwide Interrogation about the Current Management of Status Epilepticus in Germany
Source: J Clin Med. 2022 Feb 22;11(5):1171. doi: 10.3390/jcm11051171 (PMC8910893; doi:10.3390/jcm11051171)
Supplement: Supplementary file 1 [file jcm-11-01171-s001.zip › jcm-1599915-supplementary.pdf]

Kowoll et al. IGNITE Status Epilepticus Survey: a nationwide interrogation about the current management of Status epilepticus in Germany - **Questionnaire**

Survey Status epilepticus Germany

Hospital Characteristics

1. What is the size of your hospital (all departments)?

< 200 beds

200-500 beds

>500-1000 beds

>1000 -1500 beds

>1500- 2000 beds

>2000 beds

2. Your hospital is a

a. University hospital

b. Specialized or maximum care hospital

c. Basic care hospital

d. BG-Kliniken (Berufsgenossenschaftskliniken = hospitals specialized in work accidents, predominantly owned by insurances)

e. Rehabilitation clinic

f. Others

3. In which German federal state is your hospital located?

4. Which of the following departments does your hospital have?

a. Only Neurology

b. Only Neurosurgery

c. Both Neurology and Neurosurgery

5. Your intensive care unit is

a. Confined to neurological patients

b. Confined to neurosurgical patients

c. Interdisciplinary

6. Which department is your intensive care unit (ICU) associated with?

a. Neurology

b. Neurosurgery

c. Anesthesiology

d. Internal Medicine

e. Interdisciplinary

7. What is your specialty?

a. Neurology

b. Neurosurgery

c. Anesthesiology

d. Internal Medicine

e. Others

8. How many beds were operated on your intensive care unit (ICU) in 2018? In case of changing numbers due to a shortage of skilled staff give the medium value as a whole number.

9. How many neurological/ neurosurgical patients are treated on your ICU per month?

a. <1 patients
b. 1-4 patients
c. 5-10 patients
d. 11-20 patients
e. 21-30 patients
f. 31-40 patients
g. 41-50 patients
h. >50 patients

10. In general you treat

a. Children only
b. Adults only
c. Children and adults

Epidemiology of status epilepticus

11. In the treatment of patients with status epilepticus different departments/wards are involved, e.g. emergency department, intermediate care unit and intensive care unit. Which frequency do you estimate for the diagnose “status epilepticus” in your clinic in general within the year 2018? Please take all units/ departments involved into account. Please specify a whole number.

12. What is the proportion of all patients with status epilepticus in your clinic treated on your ICU?

13. Estimate the frequency of different types of status epilepticus in percent in your clinic. The numbers must add up to 100%.

a. Status with generalized tonic-clonic seizures
b. Non-convulsive status epilepticus
c. Focal status epilepticus
d. Absence status
e. None of the categories

14. Estimate the frequency of different stages of status epilepticus in percent in your clinic in 2018. The numbers must add up to 100%.

a. Responsive (remittent after 1. therapy)
b. Established (remittent after 2. therapy)
c. Refractory (remittent after >2 therapies)
d. Super-refractory (remittent under anesthetics after >24h)

Diagnostic tools on the intensive care unit

15. Is continuous EEG-monitoring available on your intensive care unit in addition to repetitive EEG?

a. Yes

b. No

c. Continuous EEG with additional video monitoring

16. When you record repetitive EEG, who is usually responsible for the hookup (multiple answers possible)?

a. Medical technical assistants
b. Doctors
c. Nurses of the ICU
d. Others

17. How is the availability of repetitive EEG on your NICU at <u>weekdays</u>?

a. During core times
b. Partial off-time availability
c. Twenty-four hours

18. How is the availability of repetitive EEG on your NICU at <u>weekends</u>?

a. Not available
b. Certain time slots during day time
c. Twenty-four hours

19. Are there tools for external EEG analysis for the supervising consultant?

a. Yes
b. No
c. Partially

20. Which phrases about standards for status epilepticus apply to your clinic?

a. We provide a written in-house standard with regular adherence.
b. We provide a written in-house standard with partial adherence.
c. We do not provide a written in-house standard, but we follow similar approaches.
d. We have no standard, treatment depends on the responsible physician.

21. Substance groups mainly used in Status epilepticus are benzodiazepines, antikonvulsives and sedatives. Please specify your preferred rankin order for status with generalized tonic-clonic seizures.

a. Anticonvulsants
b. Benzodiazepins
c. Sedatives

22. Please specify your preferred rankin order for non-convulsive status.

a. Anticonvulsants
b. Benzodiazepins
c. Sedatives

23. Please specify your preferred rankin order for focal status.

a. Anticonvulsants
b. Benzodiazepins
c. Sedatives

24. Which is the benzodiazepine of first choise in your clinic?

a. Lorazepam
b. Midazolam
c. Diazepam
d. Clonazepam
e. Others
f. No 1.choice benzodiazepine

25. Which is the anticonvulsant of first choise in your clinic?

a. Lacosamid
b. Levetiracetam
c. Phenobarbital
d. Phenytoin
e. Valproat
f. No 1.choice anticonvulsant

26. Which is the sedative of first choise in your clinic?

a. Propofol
b. Midazolam
c. Ketamin/Midazolam
d. Thiopental
e. Isofluran
f. Others
g. No 1.choice anticonvulsant

27. **is your preferred rankin order of anticonvulsants in refractory status (except for Absence-Status)?**
    a. Lacosamid
    b. Levetiracetam
    c. Phenobarbital
    d. Phenytoin
    e. Valproat

28. **Which is your preferred rankin order of sedatives in refractory status (except for Absence-Status)?**

a. Propofol
b. Midazolam
c. Ketamin/ Midazolam
d. Thiopental
e. Isofluran

29. **Which of the following therapies are established at your ICU in refractory status (multiple answers allowed)?**

    a. Immunotherapies (steroids, apheresis, immunsuppressiva…)
    b. Ketogenic diet
    c. Electroconvulsive therapy
    d. Hypothermia
    e. Epilepsy surgery
    f. Magnesium
    g. None

30. **Which answer applies most? If you use sedatives in super-refractory status epilepticus, …**
    a. we always aim for burst-suppression-anaesthesia under EEG control.
    b. we primarily aim for a cessation of clinical seizure activity without consideration od EEG.
    c. we do not use sedatives in status epilepticus.

31. **What is usually the duration of a first cycle with a sedative in ordert o terminate status epilepticus?**
    a. Less than 24 hours
    b. Between 24 and 48 hours
    c. Longer than 48 hours
    d. We do not use sedatives in status epilepticus.

32. **Which answer applies most when using sedatives for status epilepticus? What is your next step when the first cycle fails?**
    a. You repeat the cycle with the same sedative.
    b. You change the sedative and repeat anesthesia.

c. Instead of repeating anesthesia you change to another method.
d. We do not use sedatives in status epilepticus.

33. What is usually the duration of a second cycle with a sedative?

a. Less than 24 hours
b. Between 24 and 48 hours
c. Longer than 48 hours
d. We do not attempt a second cycle with a sedative.
e. We do not use sedatives in status epilepticus.

34. If you used sedatives for status epilepticus, what was the estimated termination rate after the first cycle in percent?

35. If you used sedatives for status epilepticus, what was the estimated termination rate after the second cycle in percent?

36. How often do you transfer patients with status epilepticus to another hospital?

a) We do not transfer patients with status epilepticus to another hospital in the acute phase.
b) We sometimes transfer patients with refractory oder super-refractory status epilepticus to another hospital.
c) We often transfer patients with refractory oder super-refractory status epilepticus to another hospital.
d) We often transfer patients with status epilepticus to another hospital independently of the stage.

37. If you have to transfer patients with status epilepticus, what is the exact procedure

a) There are standardized transfer structures with a cooperating clinic analogous to neurovascular emergencies.
b) There are general commitments for the transfer with a cooperating clinic, but every case has to be coordinated individually.
c) There are several eligible clinics, a transfer is organized upon request after individual consultation.
d) We do not transfer patients with status epilepticus.

Outcome and self-assessment

38. Are follow-up interviews common after the acute phase of status epilepticus?

a) Yes
b) No
c) Unknown
d) Irregularly.

39. Are you satisfied with the current care situation of status epilepticus?

a) Very satisfied.
b) Rather satisfied.
c) Rather unsatisfied.
d) Unsatisfied.

40. How confident do you feel about the treatment of status epilepticus?

a) Very confident.
b) Rather confident.
c) Rather unconfident.
d) Unconfident.

41. Is there a particular problem or issue in the treatment of status epilepticus which you would like to address?

Free text answer

Continuous EEG-Monitoring

42. Are there opportunities of simultaneous Video-EEG-monitoring on your ICU?

a. Yes
b. No

43. How many recording channels are available for continuous EEG-Monitoring?

2 4 6 8 10 12 14 16 18 21 >21

44. **How many recording channels do you usually employ for continuous EEG-Monitoring**?

2 4 6 8 10 12 14 16 18 21 >21

45. If you monitor with continuous EEG on your ICU, how long is usually the recording time?

a) <24 h
b) 24-48 h
c) >48 h

46. Who does usually perform the analysis of the continuous EEG-Monitoring?

a) Doctors from the EEG lab
b) Doctors on the ICU
c) Doctors from the EEG lab and the ICU to a comparable degree
d) Others

47. How frequently do you document the analysis of your cEEG by a written analysis?

a. > once a day
b. once a day
c. < once a day
d. No regular written analysis

48. When using continuous EEG monitoring on your ICU, how do you analyse it?

a. Only on a visual basis
b. Only by quantitative analysis tools
c. Both on a visual basis and by quantitative analysis tools

49. Which quantitative analysis tools do you employ?

a) Compressed spectral array (CSA)
b) Density spectral array (DSA)
c) Color density spectral array (CDSA)
d) Others
e) No quantitative analysis tools
